# Two new species of *Henckelia* (Gesneriaceae) from Southeastern Yunnan, China

**DOI:** 10.3897/phytokeys.130.33988

**Published:** 2019-08-29

**Authors:** Lei Cai, De-Tuan Liu, Pin Zhang, Zhi-Ling Dao

**Affiliations:** 1 Yunnan Key Laboratory for Integrative Conservation of Plant Species with Extremely Small Populations, Kunming Institute of Botany, Chinese Academy of Sciences, Kunming 650201, Yunnan, China; 2 Key Laboratory for Plant Diversity and Biogeography of East Asia, Kunming Institute of Botany, Chinese Academy of Sciences, Kunming 650201, Yunnan, China; 3 University of Chinese Academy of Sciences, 100049 Beijing, China

**Keywords:** Gesneriaceae, *
Henckelia
*, new taxon, China, flora of Yunnan

## Abstract

Two new species of Gesneriaceae, *Henckelia nanxiheensis* Lei Cai & Z.L.Dao, **sp. nov.** and *H. multinervia* Lei Cai & Z.L.Dao, **sp. nov.** from southeastern Yunnan, China, are described with color photos. The diagnostic characters of the two new species, together with photographs, detailed descriptions, distribution and habitat, as well as comparisons with morphologically similar species, are also provided.

## Introduction

The circumscription of the genus *Henckelia* Spreng. (Gesneriaceae) has been redefined by Weber et al. based on molecular and morphological evidence (Weber and Burtt 1998; Weber et al. 2011). *Henckelias.l.* consists of more than 60 species, mainly distributed across south and east Asia and the adjacent Himalayan regions (Weber et al. 2011; Manudev et al. 2012; Janeesha and Nampy 2015; Sinha and Datta 2016; Ranasinhe et al. 2016; Krishna and Lakshminarasimhan 2018; Sirimongkol et al. 2019). Southwest China harbors a high diversity of *Henckelia*, and more than 20 species of *Henckelia*, which were originally described under the names of *Hemiboeopsis* W.T. Wang and *Chirita* Buch.-Ham. ex D. Don, are recorded from China (Wang et al. 1990, 1998; Weber et al. 2011; Li and Wang 2005; Möller et al. 2016; Xu et al. 2017).

During our field investigation in southeastern Yunnan in 2016, several unusual species of Gesneriaceae caught our attention. After careful study of relevant specimens and taxonomic publications from the adjacent regions (Wang et al. 1990, 1998; Li and Zhu 2010; Weber et al. 2011; Middleton et al. 2013; Möller et al. 2016; Xu et al. 2017; Sirimongkol et al. 2019), we concluded that these plants represent two new members of *Henckelia*. Descriptions and illustrations of the new species are presented here; the morphological characters are also compared with those of closely related species.

## Taxonomic treatments

### Henckelia nanxiheensis

Taxon classificationPlantaeLamialesGesneriaceae

Lei Cai & Z.L.Dao sp. nov.

25764DBCC3DD5B939C11F4C80D09460B

urn:lsid:ipni.org:names:60479349-2

Figures 1 , 2

#### Diagnosis.

*Henckelia nanxiheensis* is morphologically similar to *H. auriculata* (J.M. Li & S.X. Zhu) D.J. Middleton & Mich. Möller in the shape, color and size of the flower; it also resembles *H. ceratoscyphus* (B.L. Burtt) D.J. Middleton & Mich. Möller in the indumentum characteristics of the whole plant and hornlike apex of the calyx, but differs from them both in the shape of leaf blade, calyx lobes, size of corolla, indumentum of pedicel and pistil.

#### Type.

China. Yunnan: Pingbian County, Baiheqiao Town, Dujiao, 22°55'N, 103°51'E, 255 m a.s.l., on the surface of rocks in the shaded valleys, in flower, 18 Mar 2017, *Lei Cai CL035* (holotype: KUN!, isotypes: KUN!).

#### Description.

Perennial; stem extremely short, usually less than 1 cm. Rhizome 0.5–1.0 cm long, internodes inconspicuous. Leaves basal, opposite; petiole 2–11 cm long, ca. 1 mm in diameter, base 3 mm wide, densely pubescent; leaf blade oblong to oval, 4–20 × 1.5–5.5 cm, herbaceous, adaxially densely puberulent, eglandular, abaxially puberulent and densely along veins, base oblique or asymmetrical, margin serrate, apex acute; lateral veins 5–12 on each side of midrib, conspicuous on both sides. Cymes 3–10 per plant, each 1–4-flowered; peduncle 1–2 cm long, pubescent; bracts 2, free, ovate to oval, ca. 3–5 × 2–3 mm, pubescent both sides, margin entire, apex acute. Pedicel 0.8–2.5 cm long, pubescent. Calyx 8–10 mm long, 5-parted to 3/4 of the length; lobes equal, lanceolate to long-triangular, 6–8 mm long, outside pubescent, inside glabrous, apex attenuate, horn-like 3–5 mm long. Corolla ca. 2 cm long, white to pale blue, with light purple stripes inside, abaxial lips purple, outside sparsely puberulent, inside glabrous; tube slightly curved, narrow at base, ca. 10–13 × 2.0–4.5 mm; adaxial lobes 2, orbicular, ca. 4 mm in diameter; abaxial lobes 3, orbicular, ca. 5 mm in diameter. Stamens 2, anthers adaxially fused, ca. 1.5 mm long, sparsely puberulent; filaments ca. 4.5 mm long, S-shaped, inserted ca. 2.5 mm from the base, glabrous below, extremely sparsely glandular-puberulent apically; staminodes 3, green, ca. 0.5 mm long, inserted ca. 3 mm from base. Disc ca. 0.5 mm high, inconspicuous. Pistil 1.1–1.3 cm long; ovary long-elliptic, 3–4 mm long, densely pubescent; style linear, ca. 8 mm long, glandular puberulent; stigma flabellate, ca. 2 mm long, 2-lobed. Fruit unknown.

**Figure 1.**
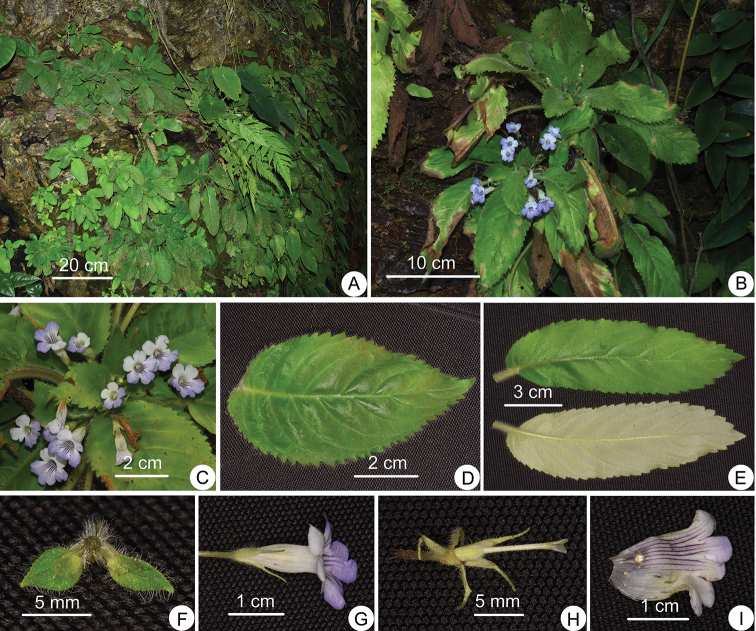
*Henckelia nanxiheensis* Lei Cai & Z.L.Dao, sp. nov. **A** Habitat **B** plants with flowers **C** front view of flowers **D** adaxial leaf surface **E** adaxial and abaxial leaf surfaces **F** bracts **G** side view of flower **H** pistil, disc and calyx **I** opened corolla. All photographs by Lei Cai.

**Figure 2.**
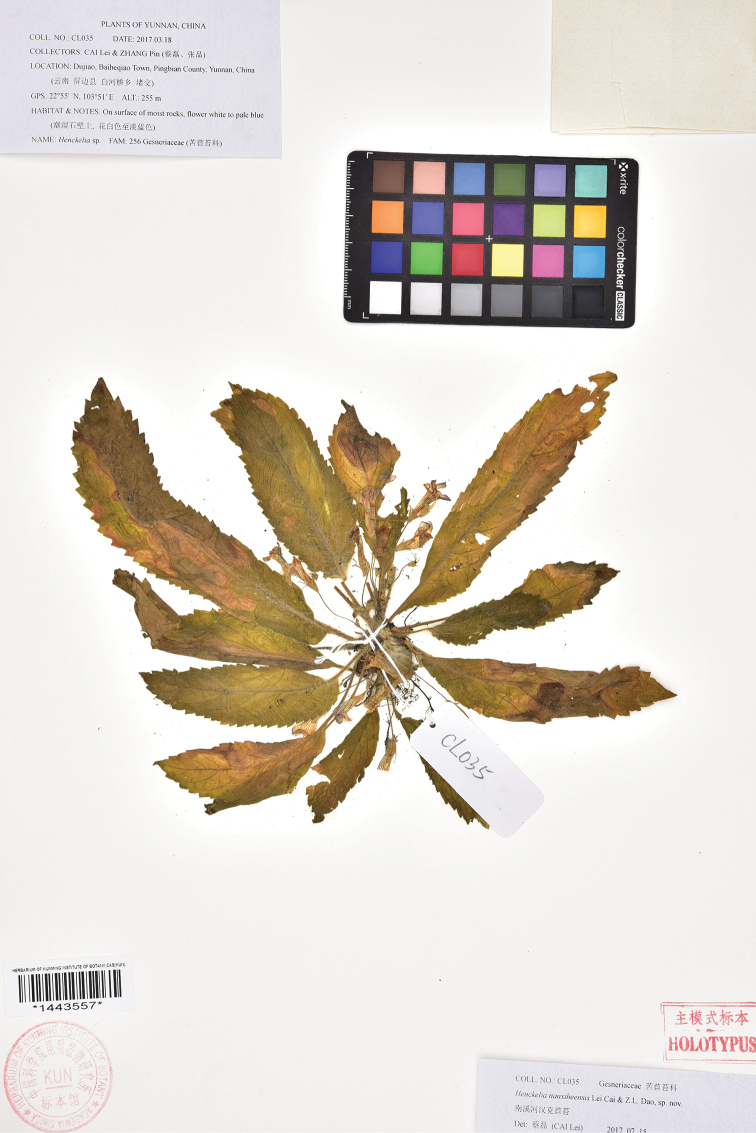
Holotype of *Henckelia nanxiheensis* Lei Cai & Z.L.Dao (KUN-1443557).

#### Phenology.

Flowering from March to April.

#### Etymology.

The specific epithet ‘*nanxiheensis*’ refers to the type locality in the regions near the Nanxi River, which is a tributary of the Red River.

#### Vernacular name.

The Chinese mandarin “nan xi he han ke ju tai” (南溪河汉克苣苔)

#### Distribution and habitat.

The new species is currently known only from the type locality with a small population of ca. 150 individuals growing on moist rocks of the entrance to the valley near the banana plantation. The population of *Henckelia nanxiheensis* was first discovered in 2014 (without flowers) by Lei Cai; individuals in blossom were collected in 2017. The disturbance of the population has been observed with ca. 15% loss of the original habitat, due to human activities which are not limited to the expansion of banana plantations. An urgent preservation scheme is required to rescue this species from further disturbances or wipe-out from the type locality.

#### Notes.

This new species most closely resembles *Henckelia auriculata* in the shapes of the flower and calyx. However, it differs from the latter in the oblong to oval leaf blade with oblique base and serrate margin (vs. rotund to elliptical with auriculate base and shallowly serrate to coarsely dentate margin); the calyx divided 3/4 of the length of calyx (vs. calyx parted to base); the flower lips white to pale blue adaxially and purple abaxially (vs. entirely white); the ovary densely pubescent and the style glandular puberulent (vs. densely but eglandulate hairy pistil). It is also similar to *H. ceratoscyphus* in the indumentum characteristics and hornlike apex of the calyx, but differs from having leaf blade oblong to oval (vs. narrowly to broadly elliptic), smaller corolla ca. 2.2 cm long (vs. 4.5 cm long), slightly curved tube (vs. tube funnelform), equal size of 3 staminodes ca. 0.5 mm long (vs. central one ca. 1.5 mm long, laterals ca. 6 mm long); the ovary densely pubescent and the style glandular puberulent (vs. puberulent pistil).

### Henckelia multinervia

Taxon classificationPlantaeLamialesGesneriaceae

Lei Cai & Z.L.Dao sp. nov.

DB7AD18F547F5A88AE04EF5F5DE01DD2

urn:lsid:ipni.org:names:60479350-2

Figures 3 , 4

#### Diagnosis.

*Henckelia multinervia* is similar to *H. ceratoscyphus* (B.L. Burtt) D.J. Middleton & Mich. Möller in the shape, color and size of the flower, the indumentum of leaf blade and the horn-like calyx apex, but it can easily be distinguished from the latter by the shape of leaf blade and distinct venation pattern, smaller horn-like calyx apex, the indumentum of corolla and style and smaller stigma.

#### Type.

China. Yunnan: Hekou County, Laofanzhai Town, Jinzhuliang village, Shiqiao, 22°47'N, 103°49'E, 442 m a.s.l., on moist rocks in rainforest valley along the bank of the stream, in flower, 3 Apr 2018, *G.L. Zhang CL2018008* (holotype: KUN!, isotypes: KUN!).

**Figure 3.**
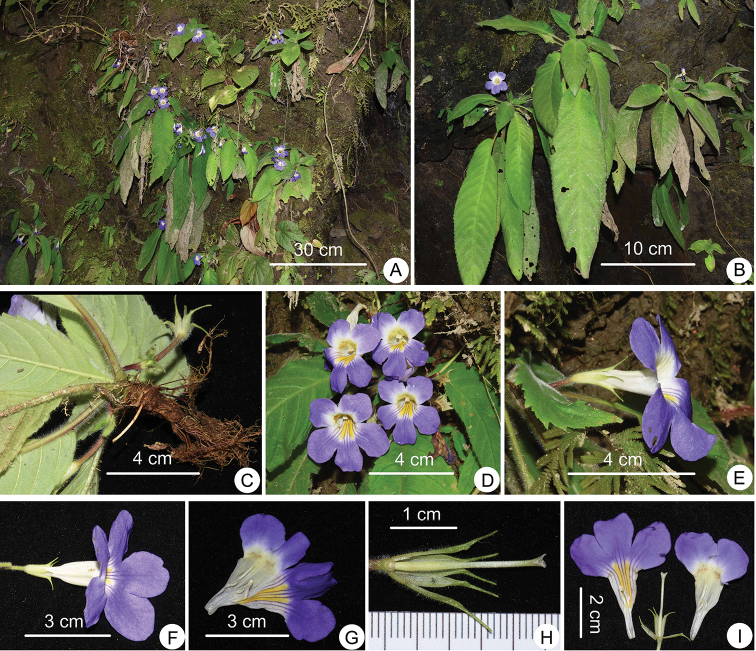
*Henckelia multinervia* Lei Cai & Z.L.Dao, sp. nov. **A** Habitat **B** plants with flowers **C** rhizome, petiole and abaxial leaf surface **D** front view of flowers and adaxial leaf surface **E, F** side view of flowers **G** opened corolla **H** pistil, disc and calyx **I** opened corolla and pistil with calyx. All photographs by Lei Cai.

**Figure 4.**
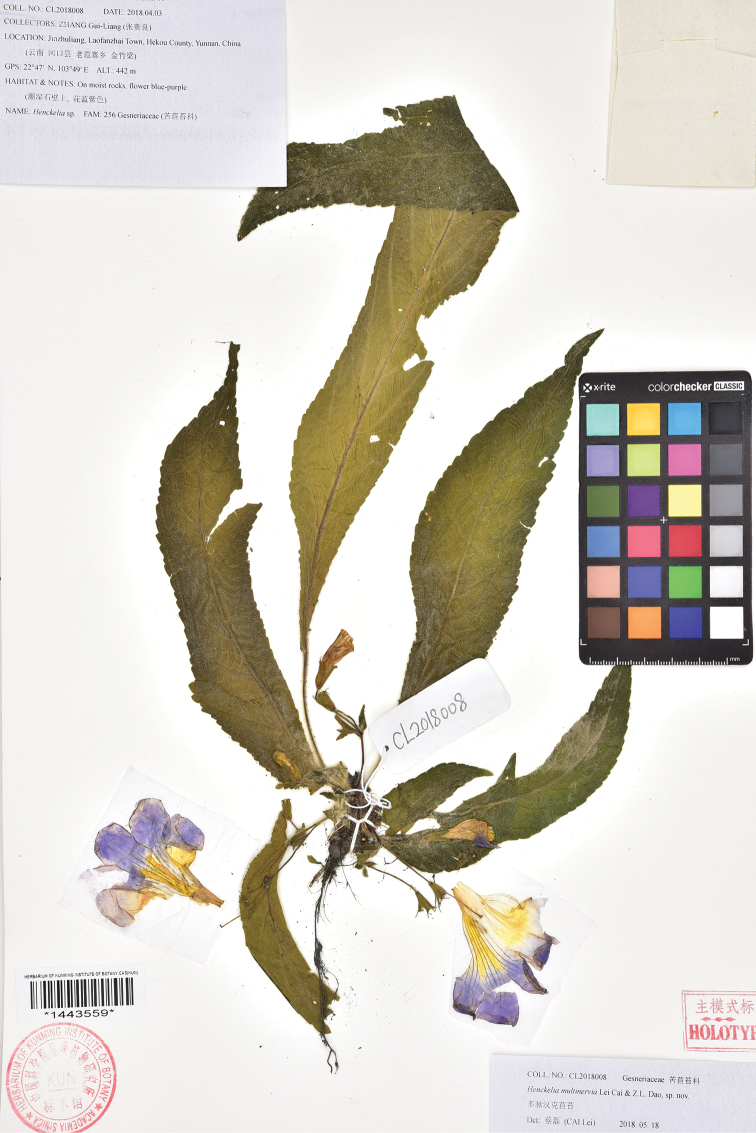
Holotype of *Henckelia multinervia* Lei Cai & Z.L.Dao (KUN-1443559).

#### Description.

Perennial, stem extremely short, usually less than 1 cm. Rhizome 0.8–5.0 cm long, internodes inconspicuous. Leaves basal, opposite; petiole 2–16 cm long, ca. 1 mm in diameter, densely pubescent; leaf blade long-elliptic to broadly lanceolate, 4–34 × 2–8 cm, herbaceous, adaxially densely puberulent, eglandular, abaxially puberulent and densely puberulent along veins, base oblique, cuneate on both sides, margin serrate, apex acute; lateral veins 12–22 on each side of midrib, conspicuous on both sides. Cymes 2–5 per plant, each 1–3-flowered; peduncle 1.2–2.5 cm long, pubescent; bracts 2, free, lanceolate, ca. 8–10 × 3–5 mm, pubescent both sides, margin entire, apex acute. Pedicel 1.5–2.5 cm long, pubescent. Calyx 1.2–2.0 cm long, 5-divided to near base; lobes equal, lanceolate to oblong, 1.2–2.0 cm long, outside pubescent, inside glabrous, apex attenuate, horn-like, 4–6 mm long. Corolla blue-purple, with two yellow stripes and several purple stripes on the abaxial lip, 3.5–5.0 cm long, outside glandular pubescent, inside glabrous; tube funnelform, outside white, ca. 2.8–4.0 × 0.2–1.5 cm; adaxial lobes 2, orbicular, ca. 1.5 cm in diameter; abaxial lobes 3, orbicular, ca. 1.8 cm in diameter. Stamens 2, anthers adaxially fused, ca. 2 mm long, puberulent; filaments 6–8 mm long, inserted ca. 1.2 cm from base, glabrous below, sparsely glandular puberulent apically; staminodes 3, central one ca. 1.5 mm long, laterals ca. 4 mm long, inserted ca. 1.3 cm from base. Disc ca. 1 mm high, shallowly 5-lobed. Pistil 2.4–2.8 cm long; ovary long elliptic, 8–10 mm long, densely pubescent; style linear, 1.5–1.8 cm long, glandular puberulent; stigma flabellate, ca. 2 mm long, 2-lobed. Old capsule (from the previous year) ca. 3.2 cm long.

#### Phenology.

Flowering from March to April; fruiting from April to May.

#### Etymology.

The specific epithet ‘*multinervia*’ is Latin, and refers to the relatively large number veins of this species.

#### Vernacular name.

The Chinese mandarin “duo mai han ke ju tai” (多脉汉克苣苔)

#### Distribution and habitat.

This species is currently known only from the type locality, where ca. 80 individuals were found on moist rocks beside a river in a rainforest valley. More field work is required to assess the conservation status.

#### Additional specimens examined.

China. Yunnan: Hekou Couty, Laofanzhai Town, Jinzhuliang village, Shiqiao, 22°47'N, 103°49'E, 442 m a.s.l., on moist rocks in rainforest valleys along stream, in flower, 11 Apr 2018, *Lei Cai et al. CL084* (KUN!); Hekou County, Laofanzhai Town, Jinzhuliang village, Shenchong, 22°47'N, 103°49'E, 485 m a.s.l., on moist rocks in the rainforest valley along streamsides, in flower, 8 Mar 2017, *G.L. Zhang ZhangGL222* (KUN!).

#### Notes.

The new species is most similar to *H. ceratoscyphus* in its habitat and floral characteristics, but can easily be distinguished from the latter by having long-elliptic to broadly lanceolate leaf blade (vs. narrowly to broadly elliptic); more lateral veins up to 12–22 pairs (vs. 7–11 pairs); the horn-like apex up to 1/4–1/3 of the length of calyx (vs. 1/2–2/3 of the length of calyx); the corolla glandular pubescent outside (vs. sparsely puberulent outside), the glandular pubescent style (vs. puberulent); and the ca. 2 mm long stigma (vs. ca. 6 mm long).

## Discussion

Based on the field observation and field notes of historical herbarium collections of *Henckelia*, most species of *Henckelia* were found on soil and rocks (non-limestone), with only four species, *H. auriculate*, *H. dimidiata* (Wall. ex C.B. Clarke) D.J. Middleton & Mich. Möller, *H. campanuliflora* Sirim. and *H. candida* Sirim. (Sirimongkol et al. 2019) were recorded from limestone areas. We believe that the habitat driven speciation is worthy of further investigation. Although a large number of new species of Gesneriaceae have been reported from China in recent years, until now, no new members of *Henckelia* have been published after the revision (Weber et al. 2011) from China, and a few new species of the genus have been described from India, Sri Lanka, Myanmar and Thailand (Manudev et al. 2012; Janeesha and Nampy 2015; Ranasinhe et al. 2016; Krishna and Lakshminarasimhan 2018; Sirimongkol et al. 2019). The two new species in this paper were all discovered in Dawei Mountain area, a locality with several different vegetation types and abundant plant diversity; therefore, we will continue to pay more attention to the Gesneriaceae species diversity in this area and adjacent region.

According to the first-hand field investigation, the two new species described here also fit the criterion of Plant Species with Extremely Small Populations (Ma et al. 2013), with unique topography and habitat, extremely limited distribution range, the population and the habitat being susceptible to human activities, e.g. collection, crop planting or deforestation. The development of a conservation strategy and action plan is urgently needed to protect the two new species.

## Supplementary Material

XML Treatment for Henckelia nanxiheensis

XML Treatment for Henckelia multinervia
